# Development of *Eimeria nieschulzi* (Coccidia, Apicomplexa) Gamonts and Oocysts in Primary Fetal Rat Cells

**DOI:** 10.1155/2013/591520

**Published:** 2013-06-19

**Authors:** Hong Chen, Stefanie Wiedmer, Sacha Hanig, Rolf Entzeroth, Michael Kurth

**Affiliations:** Institute of Zoology, Technische Universität Dresden, Helmholtzstraße 10, 01062 Dresden, Germany

## Abstract

The *in vitro* production of gametocytes and oocysts of the apicomplexan parasite genus *Eimeria* is still a challenge in coccidiosis research. Until today, an *in vitro* development of gametocytes or oocysts had only been shown in some *Eimeria* species. For several mammalian *Eimeria* species, partial developments could be achieved in different cell types, but a development up to gametocytes or oocysts is still lacking. This study compares several permanent cell lines with primary fetal cells of the black rat (*Rattus norvegicus*) concerning the qualitative *in vitro* development of the rat parasite *Eimeria nieschulzi*. With the help of transgenic parasites, the developmental progress was documented. The selected *Eimeria nieschulzi* strain constitutively expresses the yellow fluorescent protein and a macrogamont specific upregulated red tandem dimer tomato. In the majority of all investigated host cells the development stopped at the second merozoite stage. In a mixed culture of cells derived from inner fetal organs the development of schizont generations I-IV, macrogamonts, and oocysts were observed in crypt-like organoid structures. Microgamonts and microgametes could not be observed and oocysts did not sporulate under air supply. By immunohistology, we could confirm that wild-type *E. nieschulzi* stages can be found in the crypts of the small intestine. The results of this study may be helpful for characterization of native host cells and for development of an *in vitro* cultivation system for *Eimeria* species.

## 1. Introduction

The parasites of the genus *Eimeria* can be found in almost all vertebrates and some species are of high economical relevance as they can cause coccidiosis in livestock.

*Eimeria* parasites are known to have a stringent stage progression in their life cycles in the host animals. They develop mainly in the intestinal tract, but some species are also found in the liver (rabbit) or kidney (goose). To study all stages of the parasites, it is helpful to have the complete development *in vitro. *In primary cells, only a few *Eimeria* species are able to form gametocytes or complete their life cycles *in vitro* [1]. Often, this is only possible if merozoites are used for infection [2–4].

For rodent *Eimeria* species, which are frequently used as model organisms, a gametocyte or oocyst development was not achieved thus far. In previous studies with the rat parasite *Eimeria nieschulzi,* the stage development *in vitro* did not exceed the second merozoite stage [5–7]. Whereas, up to four schizont generations are found in the host before an *in vivo* gamont development occurs [8, 9]. In a study by Tilley and Upton [10], the development of four schizont generations of *Eimeria nieschulzi* in a permanent cell line under reducing conditions was described. Their results are discussed in this work.

In this study, the progress of the *in vitro* development of *Eimeria nieschulzi* was investigated using primary tissue cells from fetal rats and compared to permanent cell lines. For better documentation, a transgenic (yellow fluorescent protein; YFP expressing) *Eimeria nieschulzi* strain with an additional gamogony specific reporter (tandem dimeric tomato (TDT)) gene expression [11] was used. The TDT expression served as an indicator for successful development up to the gametocyte stage and beyond. Furthermore, we confirmed the localization of wild-type *E. nieschulzi *parasites in the crypts of the small intestine by immunohistology and compared our *in vitro* data in detail with the data in the literature.

## 2. Material and Methods

### 2.1. Cell Culture

Pregnant rats (Crl : CD (SD)) were euthanized at day 16–18 of gestation. The fetuses were extracted and decapitated prior to further usage. The decapitated fetuses were washed in cold (4°C) Dulbecco's Modified Eagle's Medium (DMEM) to remove blood. The fetuses were opened ventrally, and the inner organs were extracted. Liver, kidney, and intestine were separately collected and one assay was performed with all inner organs together as fetal mix (FM). All explanted tissues were washed twice with 37°C preheated DMEM, minced into small pieces, and ground through a sieve (mesh size 0,5 mm) with a plunger. The cell/cell conglomerate suspension was transferred to a centrifuge tube and washed once with completed DMEM (37°C) and transferred into completed preheated DMEM (with 10% FBS, 2% L-glutamine, 2% penicillin/streptomycin, 1% HEPES, and 1% sodium pyruvate). The FM-suspension was seeded in IBIDI *μ*-slides with fibronectin (IBIDI), gelatin [12], Matrigel-coating (60 *μ*L/well according to the manufacturer's recommendations) as well as in uncoated slides. For primary liver and kidney cells, fibronectin coated IBIDI *μ*-slides were used. Primary intestinal cells were seeded into IBIDI *μ*-slides, coated with fibronectin or gelatine, as well as uncoated slides. Quality evaluation of the parasite development *in vitro* was performed by comparing the following permanent cell lines: IEC6 (rat intestinal), VERO (green monkey kidney), ST (swine testicular), and IPEC-J2 (porcine small intestinal epithelial cell) which were routinely cultivated in completed DMEM. For infection with parasites, 100.000–150,000 of these cells were seeded in IBIDI *μ*-slides per well. Prior to inoculation with parasites, permanent cells were incubated about 3-4 h and primary cell about 24 h at 37°C and 5% CO_2_ atmosphere.

### 2.2. Parasites and Infection of Cells

Oocysts of the passages P2 and P3 of the previously described transgenic *Eimeria nieschulzi* [11] were used in this study. Sporozoites were excysted and purified as previously described [13] and resuspended in cell culture medium (DMEM, completed). For infection of the cells, 20.000–50.000 sporozoites were incubated together with the cells in the *μ*-slide at 37°C and 5% CO_2_ atmosphere. Every day, the cell culture medium (DMEM) was partially (50%) removed and replaced with fresh medium twice.

### 2.3. Immunohistology and Observation of Living Intestinal Parasite Stages

Rats (CD) were infected orally by gavage with 500.000–1.000.000 sporulated oocysts of *E. nieschulzi* and euthanized at different time points after inoculation with the oocysts. The exact time points can be found in the description of Figure 3. Living parasite stages were observed in small bowel tissue smears in a *μ*-slide.

For histology, the small intestine was cut into pieces of 0.5 cm length and fixed in 4% paraformaldehyde/1xPBS (pH 7.4) for 24 h at 4°C. After washing in 1xPBS and dehydration in a graded series of ethanol, the tissue pieces were embedded in paraffin. Sections of 5 *μ*m were cut with a rotary microtome (Leica RM2125RT). The sections were transferred to slides and the paraffin was removed by Roti-Histol (Co. Roth). The antigen retrieval was performed with 0.1 M sodium citrate buffer pH 6.0 for 30 min at 90°C. If HRP-linked secondary antibodies were used, endogenous peroxidase was blocked by incubation with 1% H_2_O_2_ in 1xPBS about 15 min. After blocking of unspecific antigens with 20% normal goat serum (NGS) and 0.05%  Tween in 1xPBS about 1 h at room temperature, the sections were incubated with hybridoma supernatant of the B1C4 clone [14] for about 2 h at room temperature (1 : 10 dilution in 20% NGS/0.05% Tween/1xPBS). After the removal of the primary antibody, the slides were incubated for 1 h at room temperature with the secondary antibody diluted in 20% NGS/0.05% Tween/1xPBS (FITC conjugated anti-mouse IgG, Co Sigma Aldrich F6257 1 : 100, or HRP conjugated anti-mouse IgG 1 : 200, Co Sigma Aldrich A9044). Fluorescence probed sections were washed, counterstained with DAPI (1 *μ*g/mL) about 10 min, and washed three times about 10 min prior to mounting. The sections labelled with Peroxidase (HRP) probes were equilibrated in 0.05 M Tris buffer pH 7.6 about 5 min and further incubated in DAB/H_2_O_2_ staining solution (5 mg 3,3′-Diaminobenzidine and 5 *μ*L H_2_O_2_ in 10 mL H_2_O). The staining was performed about 20 minutes. After removing all staining solutions, the sections were mounted in Mowiol/DABCO according to the manufacturer's instructions. Control experiments were performed with infected tissue but 1xPBS instead the B1C4 antibody.

### 2.4. Microscopy

Images were taken using the Zeiss Axiovert 100 microscope combined with the Olympus F-viewII monochrome CCD-camera and the image processing software Cell F (Olympus) from 24 h–270 h, inoculation (p.i.). The fluorescence of intracellular as well as extracellular parasitic stages was observed directly in the *μ*-slides with FITC filter set (Ex. D480/30) for the yellow fluorescent protein (YFP) and with a rhodamine filter set (Ex. 560/40) for tandem dimer tomato (TDT). For the visualization of the autofluorescence of the oocyst wall, the DAPI filter set (excitation 365 nm, emission long pass 420 nm) was used. The reproduction of images was performed comparable to the microscopic view according to the filter set dependent emission wavelength. Capturing of the peroxidase probed sections was performed with the Olympus C7070 camera combined with the Olympus C5060-ADU ocular adapter or with the monochrome FviewII and CellF software.

## 3.  Results

In the permanent cell lines IEC6, VERO, IPEC-J2, and ST, as well in primary liver, kidney, and intestinal cells the development stopped after formation of the second schizonts, respectively, merozoite generation (not shown). We could not observe any further development until 268 h p.i. in these mentioned cells, and thereby, we confirm the results of previous studies.

Developmental stages beyond the second-generation merozoites were observed in the fetal cell mix (FM) derived from the inner organs on the coated slides, but not in the uncoated control slide. These results were confirmed by two experimenters in separate experiments. We have to remark that only in half of the performed experiments with coated slides, crypt-like organoid structures were observed. The success of development of macrogamonts and oocysts was directly connected with occurrence of these crypt-like structures. The particular experiments and the results of parasite development are listed in Table 1. 

**Figure 1:**
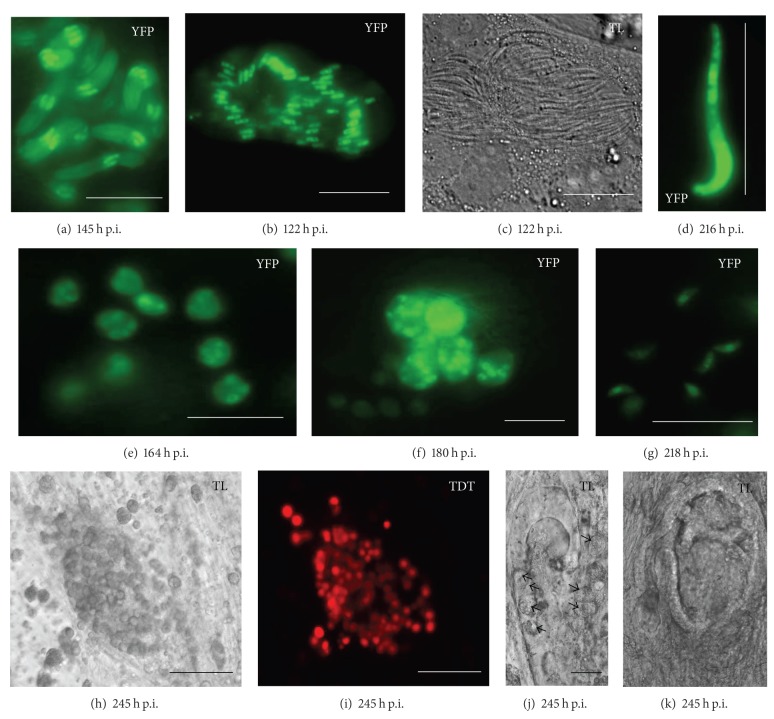
Development of the schizonts II and IV as well as clusters of macrogametocytes in organoid crypt-like structures in the fetal mix cell assay. (a) Cluster of second-generation schizonts (YFP) which can be found early from 68 h p.i. and also late at 145 h p.i. (b) and (c) probably large schizont of the second-generation; YFP (b) and transmitted light (c). (d) Probably merozoite of the second-generation (YFP) with a length of more than 20 *μ*m (22 *μ*m). Marquardt 1966 [9] and Roudabush 1937 [8] described a maximum length of 16 *μ*m. (e) Young fourth-generation schizonts (YFP) after few nuclear divisions. (f) Clustered fourth generation schizonts (YFP) after multiple cell divisions, as wells as young schizonts. (g) Fourth generation merozoites (YFP). (h) Large cluster of around 100 gamonts (transmitted light micrograph). (i) Corresponding (to (h)) fluorescence micrograph of the gamonts with macrogamont specific expressed TDT signal, YFP not shown. (j) Transmitted light micrograph with gamonts (arrows) within cells of a crypt-like structure. (k) Uninfected crypt-like structure. (a) to (g) and (j) bars = 20 *μ*m; (h), (i) and (k) bars = 100 *μ*m.

**Figure 2:**
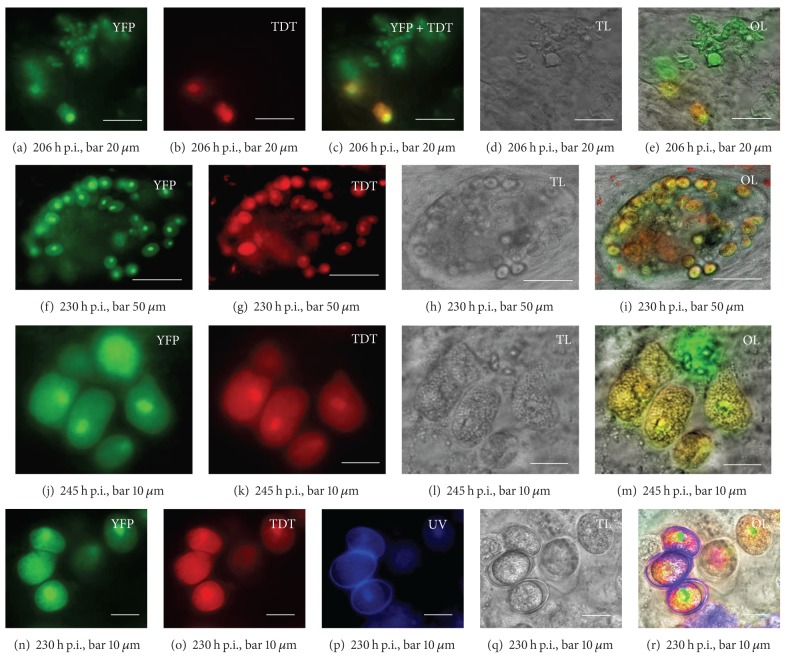
Development of macrogamonts and oocysts in primary fetal rat cells *in vitro*. (a)–(e) Transgenic schizonts IV, merozoites IV, and macrogamonts. (a) YFP is expressed in all parasite stages. (b) TDT is exclusively expressed in the macrogamonts. (c) Overlay of (a) and (b). (d) Corresponding transmitted light micrograph with low contrasted parasite stages. (e) Overlay of (a), (b), and (d) helps to identify the low contrasted gametocytes in the transmitted light micrograph and differentiating the asexual stages from the macrogamont. (f)–(i) Cluster of gamonts. (f) YFP signal (g) TDT signal. (h) Transmission light micrograph shows location of developed gamonts mainly in the periphery of a crypt like structure. An overlay of (f)–(h) is shown in panel (i). (j)–(m) Macrogamont stages adjacent to a mature schizont IV, the YFP (j) is expressed in all parasitic stages, the TDT (k) in the macrogamonts. The colocalization with the corresponding transmitted light micrograph (l) is shown in (m). (n)–(r) Formation of oocysts *in vitro*. (n) YFP signals in unsporulated oocysts and gametocytes. (o) Corresponding TDT signals. (p) Developed oocysts excited by UV light showing typical autofluorescent oocyst walls. (q) Corresponding transmitted light micrograph. (r) Overlay of (n)–(q).

In the fetal cell mix, we could identify schizonts of the first- (not shown), second- (Figure 1(a)), and fourth-generation (Figures 1(e) and 1(f)), as well as free merozoites of the second (Figure 1(d)) and fourth generation (Figure 1(g)), and also gametocytes and unsporulated oocysts (Figures 1(h)–1(j), and 2). 

The third-generation schizonts and merozoites are difficult to distinguish from the second generation. Considering the literature, the large schizont in Figures 1(b) and 1(c) may belong to the second or third generation. Despite the size, the 22 *μ*m long merozoite in Figure 1(d) would belong to the second generation. This issue is explained in the following discussion paragraph.

Schizonts of the fourth generation were found clustered with up to 15 schizonts (picture details Figures 1(e) and 1(f)) from 164 h p.i. Free merozoites IV were observed (Figure 1(g)) separately or close to the gametocytes (Figures 2(a)–2(e), 2(j)–2(m)). Gametocytes show an additional red fluorescent tandem dimeric tomato signal. The TDT-protein is expressed under the microgametocyte-specific promoter of the *gam56* gene found in *Eimeria tenella* [11, 15]. The red fluorescent signal was found the earliest at 206 h p.i. The gametocytes often occurred in clusters with up to 100 cells and grew in cells which were situated in a crypt-like formation (Figures 1(h), 1(i), and 1(j)). However, gametocytes were not found in all crypt-like structures (Figure 1(k)).

If crypt-like organoids had developed up to four clusters of macrogamonts in 25–50% of the *μ*-slide, wells could be identified. The best results were obtained in one experiment with FM-cells on a fibronectin coated *μ*-slide.

Microgamonts or free microgametes were not identified with certainty in the cell culture. Notwithstanding, unsporulated oocysts with autofluorescent oocyst walls could be observed, but these oocysts did not sporulate in the cell culture slide or outside under air supply (Figures 2(n)–2(r)). In summary, the FM-cells provide the qualitatively potential to develop *Eimeria nieschulzi* sporozoites over four asexual generations and the gametocyte stage to the point of oocyst development *in vitro.* The presence of macrogametocytes in organoid crypt-like structure indicates that these cells have similar characteristics to the native host cells in the rat.

**Figure 3:**
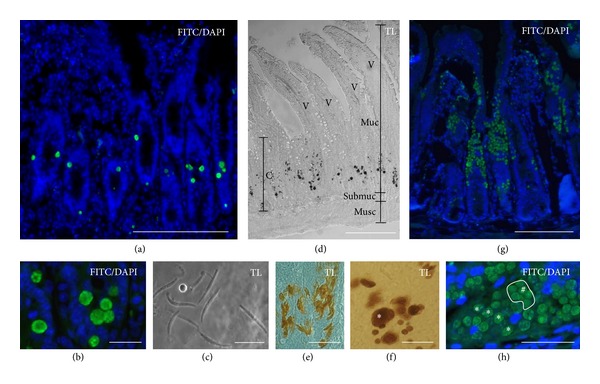
*In situ* localization of intestinal stages and third-generation merozoites. (a) FITC/DAPI overlay of B1C4 antibody probed section at 72 h p.i. Fluorescence signals are limited to the middle and upper part of the crypts but were absent in the villi. Bar 200 *μ*m. (b) FITC/DAPI overlay of B1C4 antibody probed section at 72 h p.i at higher magnification shows multinuclear schizonts. Bar 25 *μ*m. (c) Transmitted light (TL) micrograph of third-generation merozoites. At this time of infection they are characterized with a length of more than 25 *μ*m and a straight cell shape and were found in the intestine at 117 h p.i. Bar 25 *μ*m. (d) TL micrograph of B1C4 probed section at 120 h p.i., the enzymatic reaction of the secondary antibody linked peroxidase can be found in the lower and middle parts of the crypts (c). Signals are absent in the villi (v). musc: muscularis; submuc: submucosa; muc: mucosa. Bar 200 *μ*m (e) TL micrograph shows antibody derived peroxidase reaction in schizonts of the third-generation in the middle part of the crypts at 120 h p.i. The schizonts contain approximately 12 merozoites. Bar 50 *μ*m. (f) TL micrograph shows schizonts of different shapes and sizes at 120 h p.i. The asterisk designates a schizont which contained more than 12 merozoites and probably belongs to the second-generation schizont considering the stages occurred *in vitro*. Bar 25 *μ*m. (g) FITC labelled parasite stages at 144 h p.i. are visible in larger clusters in the middle and upper parts of the crypts, and at the base of the atrophied villi. Bar 200 *μ*m. (h) Higher magnifications revealed mono- and multinuclear stages (FITC/DAPI overlay). The mononuclear stages (hash key) are probably early macrogamonts and similar to them observed *in vitro*. The multinuclear stages (asterisk) are probably schizonts of the fourth generation, because the localization of nuclei is not limited to the periphery. The corresponding DAPI image can be found in Supplementary Figure 2 (see Supplementary Material available online at http://dx.doi.org/10.1155/2013/591520). Bar 50 *μ*m.

In a selected number of performed experiments we localized the wild type *E. nieschulzi in situ* in the crypt of the small intestine by immunohistology at 72, 120, and 144 h p.i. (Figure 3). The antibody B1C4 originally produced against *E. tenella *sporozoites and described 1995 by Greif and Entzeroth [14] was used for immunohistology. The antibody showed cross-reactivity to *E. nieschulzi *schizonts and gamonts.

At 72 h p.i., schizonts were detected in the middle parts of the crypts (Figures 3(a) and 3(b)). At higher magnifications, it was not possible to distinguish which schizont generation is labelled; only the visible multiple nuclei indicated schizonts. At 117 h p.i., long merozoites with a length of more than 25 *μ*m and straight cell shape in the intestine (Figure 3(c)) were identified. These merozoites belong to the third generation. At 120 h p.i., parasite stages were labelled in the middle and lower part of the crypts (Figure 3(d)). No parasites were detected in the villi. The schizonts shown in Figure 3(e) containing long merozoites belong to the third generation and were found in middle parts of the crypts. Figure 3(f) shows schizonts of different sizes in the lower part of the crypts. Marquardt [9] described large schizonts with a maximum of 12 merozoites as second or third generation. However, corresponding to the observed *in vitro* stages, we would assign them to the second-schizont-generation.

At 144 h p.i., the parasite stages were found predominantly in the middle and upper part of the crypts, as well as at the base of the villi (Figure 3(g)). At higher magnifications, it was possible to identify mono- and multinuclear stages. The mononuclear stages are probably young macrogamonts and the multinuclear stages fourth generation schizonts (Figure 3(h)). The control experiments without the primary antibodies did not show any specific signal derived by the secondary antibodies (not shown).

## 4. Discussion

In primary fetal cells, the *in vitro* development of a rodent *Eimeria* species up to the oocyst stage could be shown. As affirmed by various cell lines in this study and in previous studies, the *in vitro* development of *Eimeria nieschulzi* is limited to the second-generation merozoite [5–7]. Tilley and Upton [10] reported a development until the fourth generation *in vitro* under reducing conditions. However, *Eimeria nieschulzi* can have *in vitro* variations concerning the morphology of the schizont stages. There are large schizonts with more than 20 merozoites [11] but also small schizonts less than 10 merozoites (Figure 1(a)). Thus, it is difficult to distinguish the second schizont stage from the third schizont stage with certainty. Because *in vivo*, first- and second-generation schizonts can have delayed development up to 96 h p.i. [8, 9], the duration of the development cannot lead to concrete conclusions. Only the size of explicitly visible merozoites in or out of schizonts should be consulted for determination between second (MzII) and third-generation merozoite (MzIII), but there are further challenges concerning the interpretation of literature data. Marquardt [9] described the occurrence of MzIII beginning from 48 h p.i. with an average length of 20.8 *μ*m, 25.5 *μ*m at 72 h p.i., and 26.9 *μ*m at 120 h p.i.. At 120 h p.i., the merozoites showed a high motility while the previous smaller merozoites were nearly inactive. The schizont II, shown by Marquardt 1966 (compare Figure 8 in Marquardt 1966 [9]), is similar to* in vitro* stages at 48 h p.i. [6, 11] and we would assign them to the first-generation schizont stage. The schizont III in Figure 11 shown in the publication by Marquardt 1966 [9] is similar to that we assign to the schizont II stages (Figure 1(a)) which are visible in permanent cell lines beginning from 68 h p.i. (Figure 1(a)). A large schizont II shown by Tilley and Upton (compare Figure 7 in Tilley and Upton 1988 [10]), we would assign to schizont I stage. A schizont III shown by Tilley and Upton 1988 (compare with Figure 8 in Tilley and Upton [10]) we would assign to the schizont II stage. Rick et al. [6] also described large schizonts and long merozoites (21 *μ*m) at 73 h p.i., and assigned them to the second schizont generation. The residual tandem dimeric tomato signal, described by Hanig et al. [11], additionally supported our interpretation of the first- and second-generation stages occurring in the permanent cell culture.

Our observations around 117 h p.i. and 120 h p.i. (Figure 3) were conclusive to those described by Marquardt [9]. We could also identify large merozoites about 25 *μ*m and longer with a high motility (Figure 3(c), Supplementary Figure 3). The merozoites moved forward and backward as previously described by Marquardt [9]. Additionally, we observed movements of merozoites III with distances of more than their own cell length (Supplementary Figure 3). The model of gliding motility would prefer the forward gliding [16], and in most apicomplexan parasites this is observed in connection with banana or arch-shaped parasite cells. Backward moving is also described in *Plasmodium* in a similar dimension [17]. We think that the *E. nieschulzi* merozoites perform this by rotating on their length axis during the gliding. Otherwise, it would contradict the existing gliding motility model. The straight cell shape would support a rotation hypothesis, and we observed that the merozoites almost used the same trails during their dislocation. It is conceivable that through the high motility of this stage, a further expansion of the parasite can be accomplished. *In vitro*, we could not observe such high motile stages with more than 25 *μ*m length *in vitro,* and we are not sure if very large schizonts in Figures 1(b) and 1(c) belong to the third-generation. Third generation schizonts probably occur in very low numbers in our cell culture, which is discussed below. Marquardt [9] observed the fourth generation only at day six and described a very fast transition from merozoite III to merozoite IV. In combination with its description of merozoites III at 48 and 72 h p.i. the merozoites IV would be expected earlier. Hence, we have additional doubts concerning the early merozoites III which are probably merozoites II. To shed light on that issue, further studies with modern molecular methods should be performed.

In size and shape, the schizonts of the fourth-generation are comparable to the first-generation schizonts originated by single sporozoites. The merozoites IV are smaller than the merozoites I [8, 9]. In contrast to the isolated appearance of the first schizont stage, the fourth-generation schizonts occurred in a cluster observed from 164 h p.i. This is caused by infection of merozoites III close to their original host cell. The assumed fourth-generation schizont shown by Tilley and Upton [10] is probably a first or second-generation schizont in a delaminated host cell. Our experiments suggest that a reduced environment is not necessary for the development of *Eimeria nieschulzi in vitro.* It is assumed that the development of the third-generation schizont stage along with the invasion of the second-generation merozoites is a crucial point for the *in vitro* development. In order to invade and further develop into schizonts of the third-generation, at least the merozoites II obviously need special host cell requirements. In permanent cell lines and primary liver and kidney cells, no further development beyond the second-generation merozoite was observed. The second-generation schizont forms clusters too (Figure 1(a)), and this would also be expected for the third generation. In the nonhomogenous fetal cell mix used in this study, only a few of the merozoites II seem to find a proper host cell for invasion.

This would explain the nonoccurrence of clusters in the third-generation schizonts. Thus, parasite expansion within the third-generation schizont in cell culture would be repressed. This interpretation is supported by the occurrence of macrogamonts in only a part of the cell culture slide wells.

However, once in a proper cellular environment (crypt-like organoid), the majority of the developed merozoites III (derived from a single schizont III) can infect a new host cell. An expansion of parasitic stages occurred once more, and merozoites III developed into large clusters of schizonts IV (Figures 1(e) and 1(f)). If merozoites IV are infecting new proper host cells, they would form even larger clusters of macrogamonts and this is shown in this study.

In the natural host *Eimeria nieschulzi* directly infects crypt cells of the small intestine and can be also found in cells at the basis of the villi [8]. In other parts of the epithelium they are absent. We could confirm these original findings (Figure 3). The majority of crypt cells are known to be undifferentiated or partly differentiated. They are so-called transit-amplifying cells (TA-cells), proliferative progenitor cells derived from stem cells at the base of the crypt. Inside the villi, these former TA-cells are fully differentiated into absorptive cells and goblet cells [18]. These cells are not infected by *Eimeria nieschulzi*, but the TA-cells obviously harbor an attractor for the parasite. In the *in vitro* experiments shown here, the majority of the developed macrogamonts could be observed in crypt-like structures. This suggests that these cells have similar properties to the intestinal crypt cells in the host. These crypt-like structures were only observed in the FM-cell assay and may be based on the necessity of epithelial-mesenchymal interactions for the development and proliferation of crypt cells [19]. These mesenchymal cells were found in the FM-cell assay but not in the assay with only primary intestinal cells. This is probably the reason why intestinal cells solely do not form crypt-like organoids. Cell culture slides coated with Matrigel, fibronectin, or gelatine may be necessary for the growth of the FM-cells and the formation of crypt-like structures, while the cell culture medium seems to be less important. Nevertheless, the formation of crypt-like organoid occurred not absolutely reliable (Table 1). The FM cells are a variable undefined cell mixture with different cell content in every preparation and this can influence the cell growth. Furthermore, a high content of red blood cells seems to negatively affect the growth of the primary cells and this content alternates also in the cell preparations. Thus, in further experiments the growth of the crypt-like organoids should be aspired in a more defined manner.

The possibility to build crypt-villous structures *in vitro* from single Lgr5+ stem cells without a mesenchymal niche by stimulating the cell proliferation with external factors was shown 2009 by Sato et al. [20] in the mouse model. Their results could give further perspectives for the *in vitro* cultivation of potentially crypt cell affine *Eimeria *species. 

The molecules involved in invasion of *Eimeria* parasites, more precisely *Eimeria tenella*, are relatively well known [21, 22]. However, there is a little characterization of the surface molecules and gene expression of the natural host cells. *Eimeria *species can infect multiple cell lines, but stop their development finally after one or two schizont generations. Established cell lines seem to be only partly sufficient to explore the involved molecules for infection of host cells because they only harbour a minimum of qualities for infection and development of the parasites. It is striking that *in vivo E. nieschulzi* directly infects the cell of the crypts and passes the villous cells, whereas *in vitro* they infect several types of epithelial cells. Evidently, there are factors of the villous cells which prevent an infection and factors of the crypt cells which trigger an infection *in vivo*. Sialylated glycans on the host cell surface and MIC3 protein on the parasites side interact and may play an important role in host cell tropism of *Eimeria tenella* [23]. However, a colocalization of *E. tenella *stages and the lectin MAAII (a lectin binding to *α*2–6 or *α*2-3 sialyl linked glycan) positive cells as well as the existence of such sialylated glycans in primary chicken kidney cells, where a complete development of *E. tenella in vitro* is possible, was not shown. Therefore, it is still unclear if sialylated glycans are the most important mediators for host cell recognition. Thus, the characterization and isolation of natural host cells have to be improved to designate the host cell ligands needed for infection and development. Genetically modified *Eimeria* species are helpful for these studies.

Furthermore, it was remarkable that no microgamonts or microgametes in FM-cell culture were definitively observed, whereas macrogametocytes and oocysts were clearly developed. There are several explanations possible. First, no microgamonts developed, and no further microgametes developed. Second, microgamonts developed, but microgametogenesis of the microgametes was disturbed, for example, by serum. It would be also possible that through the three-dimensional character of the crypt-like organoid, a detection of microgamonts could not be performed by transmitted light microscopy. Only the macrogamonts have a specific marker, and microgamonts are supposed to express only the yfp fluorochrome. Our observation in small bowel smears showed only weak fluorescence signals in microgametes (not shown) and no micrographs could be taken through the rapid movement of the microgametes. Shi et al. [24] described the development of yfp-expressing microgametes of *Eimeria tenella in vitro,* but did not show any transmitted light micrographs. They mentioned that the nuclei in microgametes in immature microgamonts were not clear. We could also see only yfp expressing stages between TDT-expressing macrogamonts with no distinct nuclear signals (Supplementary Figure 1) which were regularly observed in schizont stages (Figures 1(e) and 1(f)). Also, the macrogamont typical central nuclear (Figure 1(f)) signal was absent. The question still remains if these stages are microgamonts.

Since we had not observed free microgametes and the oocysts did not sporulate, we assume no fertilization of the macrogamete took place. As the oocyst walls were formed *in vitro,* the wall formation process seems to be independent from the fertilization process. Ferguson [25] assumed that even fertilization is not obligatory for sporulation in *Toxoplasma gondii* and other Apicomplexa. Our observations do not support this speculation. More studies need to be done. Crossing experiments with different genetically modified parasites could shed light in these processes. In that manner, the genus *Eimeria* could even acts as a model for *Toxoplasma gondii *because such experiments are more difficult to accomplish in cats than in rats or chicken. The transfection technology is already established for *Eimeria *species infecting these host animals [7, 11, 26, 27] and can give the opportunity to deepen the knowledge of sexual processes in oocyst-forming Apicomplexa.

## Supplementary Material

Supplementary Figure 1: Variations of fluorescence pattern within a gamont cluster. A: The YFP fluorescence shows four parasite stages side by side. Only the exterior stages are showing a strong nuclear signal. The both internal stages do not show such a nuclear signal. B: The TDT fluorescence pattern indicates the exterior stages as macrogamonts by the gam56 promotor driven specific TDT expression. The internal stages do not show TDT expression. C: Overlay of A and B. D: Overlay of C and a transmitted light micrograph shown in E. Through the absence of TDT fluorescence and as well as nuclear signals, the internal stages can be interpreted either as young macrogamonts still without gam56 expression, or untypical schizonts without accentuated nuclear YFP signals, or even microgamonts. Shi et al. (2008) described the nuclei of microgametes in immature microgamonts as not clear. Finally the status of the shown internal stages between the exterior macrogamonts remains unclear. Bar: 20 *μ*m.
Supplementary Figure 2: DAPI fluorescence signal corresponding to Figure 3 H shows nuclei of host cells and parasitic stages at 144 h p.i. Bar 50 *μ*m.
Supplementary Figure 3: Image sequence of 31 (11 shown) micrographs illustrate the movement of a third generation merozoite in a small bowel tissue smear (148 h p.i.) The long, straight merozoites moved forward about two times of its own length and then backward about 3 times of its own length. Bar: 25 *μ*m.

## Figures and Tables

**Table 1:** Number and type of experiments performed in this study.

Cells	Coating	Cell growth	Max. parasite development
IPEC-J2 [2]	Uncoated	Monolayer	Sch II/Mz II
ST [2]	Uncoated	Monolayer	Sch II/Mz II
VERO [>2]	Uncoated	Monolayer, later multilayer	Sch II/Mz II
IEC6 [>2]	Uncoated	Monolayer	Sch II/Mz II
Prim.intestine [3] Prim.intestine [1] Prim.intestine [1]	Fibronectin Gelatin Uncoated	Monolayered fibroblast, degrading cell conglomerates, no growing organoids	Sch II/Mz II
Primary liver [2]	Fibronectin	Monolayer	Sch II/Mz II
Primary kidney [2]	Fibronectin	Monolayer	Sch II/Mz II
Primary fetal mix [3]	Fibronectin	Monolayer and cell conglomerates in two experiments (1., 2.) no growing organoids in one experiments (3.) growing crypt-like organoids	(1.) Sch II/Mz II (2.) Sch II/Mz II (3.) Mag/Ooc
Primary fetal mix [2]	Gelatin	Monolayer and cell conglomerates, in one experiment no organoids (1.) in one experiment growing crypt-like organoids (2.)	(1.) Sch II/Mz II (2.) Mag/Ooc.
Primary fetal mix [1]	Matrigel	Monolayer and growing organoids	Mag/Ooc
Primary fetal mix (3)	Uncoated	Monolayered fibroblast, degrading cell conglomerates, no growing organoids	Sch II/Mz II

[number of experiments]; (experiment number) Sch II: schizont 2nd generation, Mz II merozoite 2nd generation, Mag: macrogamont, Ooc: oocyst.
