# Supplementary material for: Development of Eimeria nieschulzi (Coccidia, Apicomplexa) Gamonts and Oocysts in Primary Fetal Rat Cells
Source: J Parasitol Res. 2013 Jun 19;2013:591520. doi: 10.1155/2013/591520 (PMC3703804; doi:10.1155/2013/591520)
Supplement: Supplementary file 1 — Supplementary Figure 1: Variations of fluorescence pattern within a gamont cluster. A: The YFP fluorescence shows four parasite stages side by side. Only the exterior stages are showing a strong nuclear signal. The both internal stages do not show such a nuclear signal. B: The TDT fluorescence pattern indicates the exterior stages as macrogamonts by the gam56 promotor driven specific TDT expression. The internal stages do not show TDT expression. C: Overlay of A and B. D: Overlay of C and a transmitted light micrograph shown in E. Through the absence of TDT fluorescence and as well as nuclear signals, the internal stages can be interpreted either as young macrogamonts still without gam56 expression, or untypical schizonts without accentuated nuclear YFP signals, or even microgamonts. Shi et al. (2008) described the nuclei of microgametes in immature microgamonts as not clear. Finally the status of the shown internal stages between the exterior macrogamonts remains unclear. Bar: 20 μm. Supplementary Figure 2: DAPI fluorescence signal corresponding to Figure 3 H shows nuclei of host cells and parasitic stages at 144 h p.i. Bar 50 μm. Supplementary Figure 3: Image sequence of 31 (11 shown) micrographs illustrate the movement of a third generation merozoite in a small bowel tissue smear (148 h p.i.) The long, straight merozoites moved forward about two times of its own length and then backward about 3 times of its own length. Bar: 25 μm. [file 591520.f1.pdf]

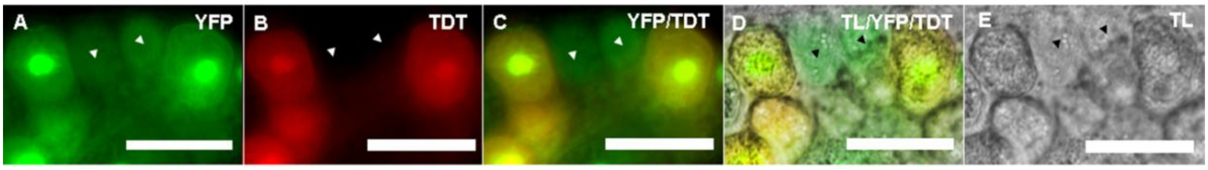

Supplementary Figure 1: Variations of fluorescence pattern within a gamont cluster. A: The YFP fluorescence shows four parasite stages side by side. Only the exterior stages are showing a strong nuclear signal. The both internal stages do not show such a nuclear signal. B: The TDT fluorescence pattern indicates the exterior stages as macrogamonts by the gam56 promotor driven specific TDT expression. The internal stages do not show TDT expression. C: Overlay of A and B. D: Overlay of C and a transmitted light micrograph shown in E. Through the absence of TDT fluorescence and as well as nuclear signals, the internal stages can be interpreted either as young macrogamonts still without gam56 expression, or untypical schizonts without accentuated nuclear YFP signals, or even microgamonts. Shi et al. (2008) described the nuclei of microgametes in immature microgamonts as not clear. Finally the status of the shown internal stages between the exterior macrogamonts remains unclear. Bar: 20 µm.

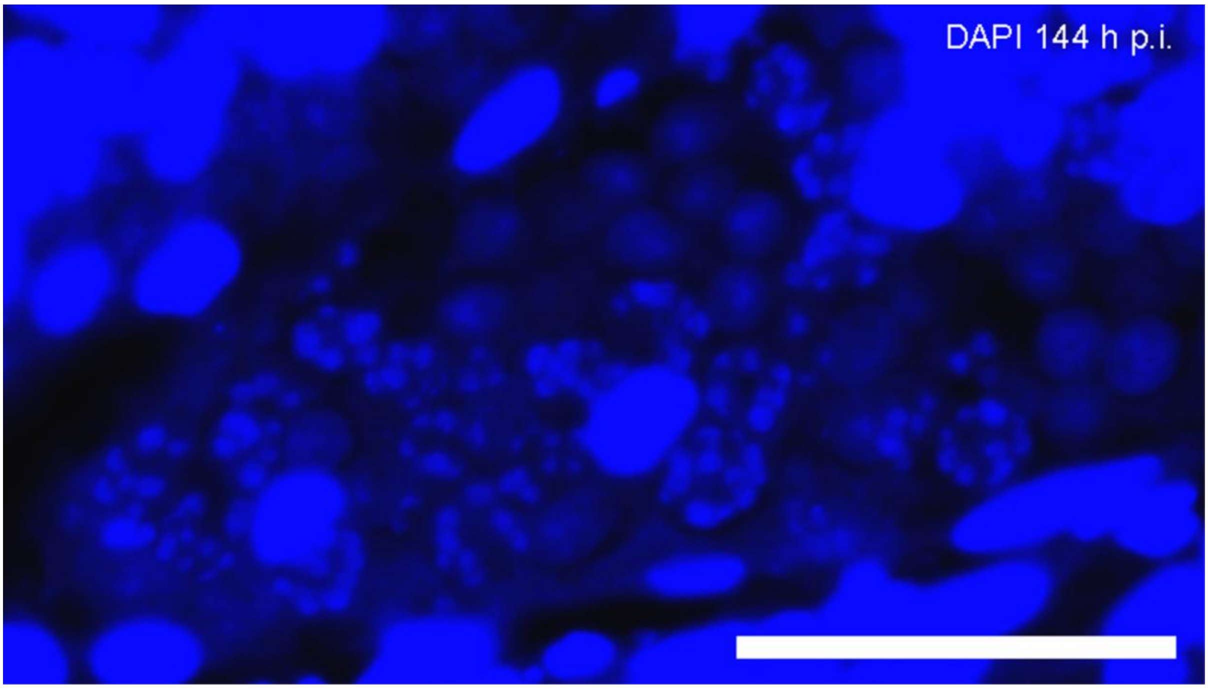

Supplementary Figure 2: DAPI fluorescence signal corresponding to Figure 3 H shows nuclei of host cells and parasitic stages at 144 h p.i. Bar 50 µm.

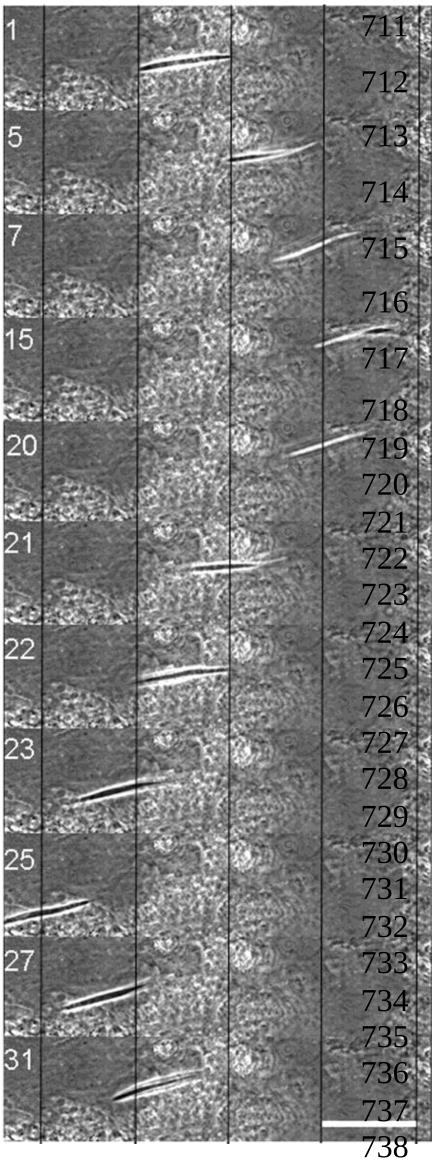

Supplementary Figure 3: Image sequence of 31 (11 shown) micrographs illustrate the movement of a third generation merozoite in a small bowel tissue smear (148 h p.i.) The long, straight merozoites moved forward about two times of its own length and then backward about 3 times of its own length. Bar: 25 µm.
